# Psychological and Social Factors Associated with Late Pregnancy Iron Deficiency Anaemia in Rural Viet Nam: A Population-Based Prospective Study

**DOI:** 10.1371/journal.pone.0078162

**Published:** 2013-10-22

**Authors:** Thach Duc Tran, Beverley-Ann Biggs, Tuan Tran, Gerard J. Casey, Sarah Hanieh, Julie Anne Simpson, Terence Dwyer, Jane Fisher

**Affiliations:** 1 Research and Training Centre for Community Development, Hanoi, Viet Nam; 2 Jean Hailes Research Unit, School of Public Health and Preventive Medicine, Monash University, Melbourne, Australia; 3 Centre for Women's Health Gender and Society, Melbourne School of Population and Global Health, The University of Melbourne, Melbourne, Australia; 4 Department of Medicine (RMH/WH), The University of Melbourne, The Royal Melbourne Hospital, Melbourne, Australia; 5 Centre of Clinical Research Excellence in Infectious Diseases (CCREID), The Royal Melbourne Hospital, Melbourne, Australia; 6 Centre for Molecular, Environmental, Genetic & Analytic Epidemiology, Melbourne School of Population and Global Health, The University of Melbourne, Melbourne, Australia; 7 Murdoch Children’s Research Institute, Royal Children's Hospital, Melbourne, Australia; Kyushu University Faculty of Medical Science, Japan

## Abstract

**Objectives:**

The aim of this study was to examine the relationships between psychological and social factors and late pregnancy IDA among pregnant women in rural Viet Nam.

**Methods:**

Pregnant women from 50 randomly-selected communes within Ha Nam province were recruited and assessed at 12 - 20 weeks gestation (Wave 1, W1). They were followed up in the last trimester (Wave 2, W2). IDA was defined as Haemoglobin < 11 g/dL and serum ferritin < 15 ng/mL. Symptoms of Common Mental Disorders (CMD) were assessed by the Edinburgh Postnatal Depression Scale-Vietnam (EPDS-V). Persistent antenatal CMD was defined as having an EPDS-V score ≥ 4 in both W1 and W2. Hypothesis models were tested by Structural Equation Modeling analyses.

**Results:**

A total of 378 women provided complete data at both W1 and W2. The incidence risk of IDA in the third trimester was 13.2% (95% confidence interval (CI): 9.8-16.7). Persistent CMD was found in 16.9% (95% CI: 13.1-20.7) pregnant women and predicted by intimate partner violence, fear of other family members, experience of childhood abuse, coincidental life adversity, and having a preference for the sex of the baby. There was a significant pathway from persistent CMD to IDA in late pregnancy via the length of time that iron supplements had been taken. Receiving advice to take iron supplements and higher household wealth index were indirectly related to lower risk of late pregnancy IDA. Early pregnancy IDA and being multi-parous also contributed to late pregnancy IDA.

**Conclusions:**

Antenatal IDA and CMD are prevalent public health problems among women in Viet Nam. The link between them suggests that while direct recommendations to use iron supplements are important, the social factors associated with common mental disorders should be addressed in antenatal care in order to improve the health of pregnant women and their infants.

## Introduction

Antenatal iron deficiency anaemia (IDA) can cause adverse perinatal outcomes, including increased risk of pregnancy-related death and preterm birth in the mother and low birthweight, and delayed cognitive development in the child [1]. Iron supplementation is a proven and effective public health intervention to reduce IDA and has been recommended for pregnant women for several decades [2]. Despite remarkable achievements worldwide in the promotion of universal antenatal iron supplementation, the prevalence of IDA among pregnant women remains high and of serious concern in low and lower-middle-income countries [1,3]. This has been attributed to several factors including poor compliance with the daily recommended supplementation regime [4-7].

Social and psychological factors may contribute to IDA and other micronutrient deficiencies. Household poverty, food insecurity, and women having a low level of education have been associated with iron deficiency and anaemia, most probably attributable to insufficient resources to purchase supplements or iron-rich foods, and lack of knowledge about iron deficiency [5,7]. Several studies have suggested a link between anaemia and/or iron deficiency and women’s psychological status [8,9]. Beard et al. found a significant correlation between IDA and higher Edinburgh Postnatal Depression Scale scores nine months postpartum in women in South Africa [8]. Postpartum depression symptoms have also been correlated with severity of anaemia in women in the USA [9] and Iran [10]. These studies were cross-sectional and therefore not able to attribute the direction of the relationship. Nevertheless, the investigators of those studies concluded that IDA preceded and contributed to common mental disorder (CMD) symptoms [8,11,12]. The potential mechanisms of this effect are not well understood, but it is hypothesized that IDA alters myelination and neurotransmitter metabolism and function, which in turn contributes to depressive symptoms including fatigue, irritability, apathy, and an inability to concentrate [13,14]. The possibility that these two conditions are reciprocally related or that the relationship acts in both directions has not been considered to date. 

There are correlations between antenatal depression and preventive health care behaviours in high-income countries. Minkovits et al. reported that children of mothers reporting depressive symptoms had fewer age-appropriate well-child visits and were behind their vaccination schedule at 24 months in the USA [15,16]. In Viet Nam, a lower-middle income country, we have shown that pregnant women with CMD are less likely to take antenatal iron supplements [17] or use iodised salt [18]. 

The aim of this study was to establish whether psychological and social are associated with late pregnancy IDA among women in rural Viet Nam. 

## Methods

This study was part of a population-based prospective study that recruited women in early pregnancy and followed maternal and infant outcomes until six months post-partum. 

### Study setting

The study was undertaken in Ha Nam, a rural province in northern Viet Nam. It has a population of 0.8 million inhabitants most of whom live in lowland rural delta areas and rely on subsistence agriculture, principally rice farming. The average annual per capita income in 2010 was USD800 and about 7.5% of people lived below the international poverty line of USD1.25 per day [19].

### Sampling and recruitment

Participants were recruited by a two-stage sampling procedure. First, an independent statistician randomly selected 50 of the 116 communes in the province. Then, all women who were 12 to 20 weeks gestation living in the selected 50 communes during the enrolment period (from December 2009 to January 2010) were eligible and invited to participate in the study [20]. 

### Data sources

Biological and psychosocial data were collected in two antenatal surveys. The first (Wave One, W1) was conducted when participants were enrolled and the second (Wave Two, W2) when they were at least 28 weeks gestation.

#### Biological data (W1 and W2)

Hemoglobin (Hb) was evaluated in the field from a finger prick blood sample, using a hemoglobinometer (HemoCue AB, Angelholm Sweden). Serum from a 3 mL venous blood sample was analysed for serum ferritin at the Pathology Services of the Alfred Hospital, Melbourne, Australia. Serum ferritin was evaluated by Chemiluminescent Microparticle ImmunoAssay performed on the Archicentre ci62000 instrument (Abbott, Illinois, USA). Criteria for IDA were Hb < 11 g/dl and serum ferritin < 15 ng/mL as recommended by WHO and UNICEF [21].

#### Psychosocial data

Psychosocial data were collected by experienced researchers in structured interviews using standardized psychometric instruments that have been locally validated and used extensively in prior research [22,23].

Common mental disorders (W1 and W2) were assessed by the Edinburgh Postnatal Depression Scale-Viet Nam Validation (EPDS) [24,25]. The EPDS includes ten fixed choice items scored from 0 - 3 which yield a total score from 0 to 30. It has been validated in rural Viet Nam against the gold-standard of psychiatrist-administered diagnostic interviews [25]. A score of four or more has optimal sensitivity (70%) and specificity (73%) to identify clinically significant symptoms of CMD in Vietnamese women who are pregnant or have recently given birth. 

Use of iron supplements (W1 and W2) was assessed in fixed-choice and open-ended questions about whether iron supplements had or had not been taken during the index pregnancy (1: yes; 0: no), the total duration of use (gestational ages of starting and stopping taking supplements, duration of temporary cessation of taking supplements), reasons not to take iron supplements (open-ended question), and whether or not advice about the use of iron supplements had been received (1: yes; 0: no) and from whom (open-ended question). 

The sociodemographic factors (W1) assessed included: age and marital, educational and occupational status. Household economic status was assessed by the World Bank method which calculates a Household Wealth Index from information about 17 household characteristics, services and durable assets [26]. Coincidental life adversity was assessed in a single question: *Apart from your pregnancy are there other experiences or aspects of your life that are worrying*? For those who answered in the affirmative it was followed by an open-ended question asking for a description of the source of worry. 

Household food security was measured by the Household Food Insecurity Access Scale [27] which includes nine questions on degree of anxiety and uncertainty about the household food supply, adequacy of food quality, and sufficiency of food intake in the past four weeks.

Reproductive health (W1) including gravidity, parity and history of spontaneous abortions, and foetal or neonatal deaths were collected by study-specific questions. 

Quality of relationship with the intimate partner (W1 and W2) was assessed using the 24-item Intimate Bond Measure (IBM) [28]. It has been translated, culturally verified and validated against study-specific questions about supportive and coercive partner behaviours in this setting and found to be meaningful and acceptable to respondents and to yield scores which correlate highly and in expected directions with measures of mood [29]. The measure yields two subscales, Care and Control. The first records care expressed emotionally as well as physically, with constructs of warmth, kindness, consideration, affection and companionship. The Control dimension identifies dominating, intrusive, critical and authoritarian attitudes and behaviours. Scores on each subscale range from 0 to 36, with higher scores being positive on Care, but negative on Control dimensions. Experiences of intimate partner violence were assessed with the pregnancy section of the WHO Multicountry study [30] on Domestic Violence survey, which identifies physical and sexual violence, and emotional abuse. 

### Procedure

Both biological and psychosocial data were collected from each participant in private rooms at commune health centres. Expert phlebotomists from the National Institute for Malariology and Entomology, Hanoi collected the blood samples, which were centrifuged in the field to separate serum. The serum was stored at -80°C, first at the National Institute for Haemotology and Blood Transfusion, Hanoi, Viet Nam and then transported on dry ice to Alfred Pathology Services, Melbourne, Australia for laboratory analysis.

Psychosocial data in W1 and W2 were collected by the same team of eight trained and closely supervised health research workers from the Research and Training Centre for Community Development, Hanoi and recorded on paper forms. The data collectors were blinded to study hypotheses. In Viet Nam collection of psychosocial data by individual interview is preferred as self-report questionnaires are unfamiliar to most people. All data were collected between December 2009 and June 2010.

### Data management and analysis

Data management and univariable analyses were performed in STATA version 11 (StataCorp LP, College Station, Texas, United States of America). Persistent CMD was defined as an EPDS score ≥ 4 [31] in both early (W1) and late (W2) pregnancy. The relative length of time taking iron supplements was calculated by the actual time the women took iron supplements divided by the duration of gestation at W2 in weeks and converted to a percentage. The intimate partner violence index was constructed by IBM care score, IBM control score, and experience of intimate partner physical, sexual and emotional abuse. A high score indicated more violence toward the woman. Household economics were assessed by a wealth index which is a proxy measure constructed from 17 household characteristics, services and durable assets [26]. A higher wealth index score indicated a better household economic situation. 

We hypothesised that maternal CMD increased the risk of iron deficiency anaemia during pregnancy via reducing compliance with antenatal iron supplementation. Structural Equation Modeling analysis (SEM) was performed in Mplus version 6 (Muthén & Muthén, Los Angeles, United States of America) to simultaneously examine the direct and indirect effect of persistent CMD on IDA at last trimester. The possible factors of CMD, IDA and a possible mediator, the length of time taking iron supplements, were included into the model. The model was estimated using weighted least-squares and the probit link function which is recommended for binary outcomes (i.e. persistent CMD and IDA at last trimester). Indirect and direct effects were calculated, tested for statistical significance. Probit coefficients were converted to odds ratios for more straightforward interpretation [32].

In order to evaluate model fit, we used Chi-Square Test of Model Fit with p values greater than 0.05 indicating a good fit, Root Mean Square Error Of Approximation (RMSEA) with values less than 0.05 indicating a good fit, and Tucker-Lewis Index (TLI) and Comparative Fit Index (CFI) with values greater than 0.95 indicating a good fit [33]. Only women who had no missing data for any of the variables were included in the SEM analysis. 

### Ethics Statement

This study was conducted according to the guidelines laid down in the Declaration of Helsinki and all procedures involving human subjects were approved by the Ha Nam Provincial Health Department Ethics Committee, the Viet Nam Medical Association Ethics and Scientific Committee and the University of Melbourne’s Health Sciences Human Ethics Sub-Committee. Written informed consent was obtained from all participants or those who could not write were able to provide a thumb print on the consent form after having had the plain language statement read to them. 

## Results

### Sample

In total 497/523 (97%) eligible women were recruited and assessed at WI. There were 78 women (16%) lost to follow-up at W2 because they had already given birth (47 women); were away from the area at the time the data collection team visited the commune (15 women); had a stillbirth prior to follow-up (7 women); or withdrew from the study (9 women). At W2, 24 women refused Hb tests and 41 women did not provide a sample of venous blood. Overall, 378 of 523 (72.3%) eligible women provided compete data and were included in the statistical model analyses. There were no significant differences between women who did or did not provide complete data in terms of sociodemographic and reproductive characteristics, exposure to gender-based violence (Table 1), IDA at W1 (2.4 vs. 2.5, p=0.9) and CMD at either W1 (38.9% vs. 48.7%, p=0.16) or W2 (28.0 vs. 29.3, p=0.87).

**Table 1:** Demographic characteristics and social circumstances of participants who provided compete and not complete data, Ha Nam province, Viet Nam in 2010.

**Characteristic**	**Complete data (N=378)**	**Not Complete data (N=119)**	**Total (N=497)**
Age (years), mean [SD]	26.2 [4.8]	25.6 [4.9]	26.0 [4.8]
Gestational age (weeks), mean [SD]			
*Wave 1*	16.6 [2.9]	17.1 [3.3]	16.8 [3.0]
*Wave 2*	33.1 [2.0]	33.0 [2.0]^a^	33.1 [2.0]
Education, No. (%)			
*Partial or complete primary school (Grades 1–5)*	68 (18.0)	24 (20.2)	92 (18.5)
*Secondary school (Grades 6–9*)	202 (53.4)	57 (47.9)	259 (52.1)
*High school (Grades 10–12)*	46 (12.2)	12 (10.1)	58 (11.7)
*Any post-secondary education*	62 (16.4)	26 (21.8)	88 (17.7)
Occupation, No. (%)			
*Farmer*	176 (46.6)	46 (38.7)	222 (44.7)
*Factory, handcraft worker or retailer*	115 (30.4)	43 (36.1)	158 (31.8)
*Government or private officer*	46 (12.2)	15 (12.6)	61 (12.3)
*Not currently engaged in income-generating activity*	41 (10.8)	15 (12.6)	56 (11.2)
Prior miscarriages/stillbirths, No. (%)	69 (18.2)	18 (15.1)	87 (17.5)
Multiparous, No. (%)	73 (19.3)	15 (12.6)	88 (17.7)
Household food insecurity, No. (%)	114 (30.1)	44 (37.0)	158 (31.8)
Lifetime experiences of intimate partner physical violence, No. (%)	49 (13.0)	15 (12.6)	64 (12.9)
Lifetime experiences of intimate partner sexual violence, No. (%)	12 (3.4)	5 (4.2)	18 (3.6)
Lifetime experiences of intimate partner emotional abuse, No. (%)	37 (9.8)	10 (8.4)	47 (9.5)
Fear of other family members, No. (%)	22 (5.9)	3 (7.3)^a^	25 (6.0)

SD – standard deviation; ^a^n=41

The prevalence of lifetime experience of intimate partner violence (13%), fear of other family members (5.9%) and household food insecurity (30%) in women included in the analysis are consistent with other prior research in this setting [17,34]. 

### Antenatal CMD and IDA

**Figure 1:**
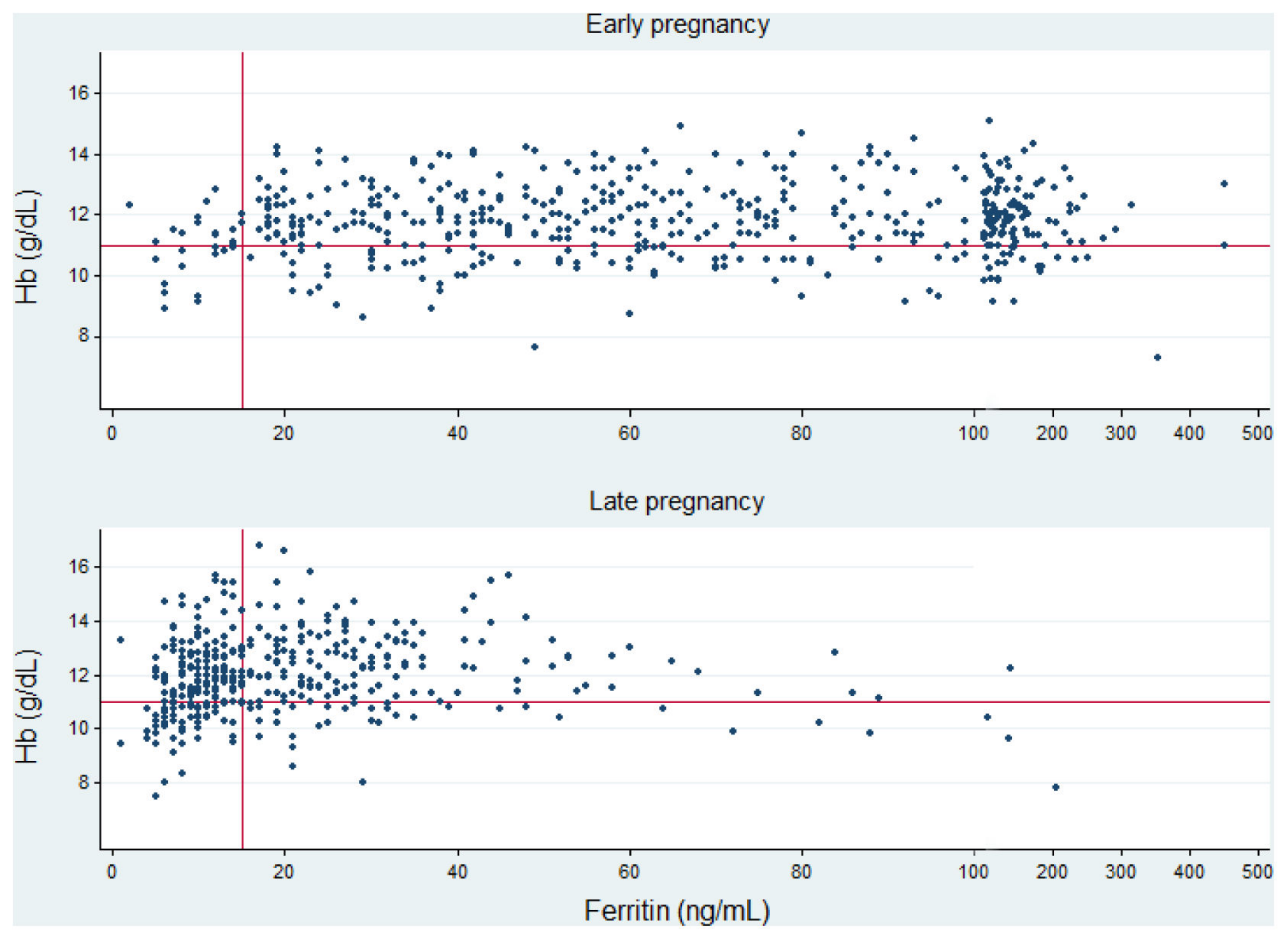
Scatter plots of ferritin and haemoglobin in early and late pregnancy in 378 women, Ha Nam province, Viet Nam in 2010. References lines for iron deficiency (ferritin < 15 ng/ml) and anaemia (Hb < 11 g/dL).

Table 2 presents the prevalence of CMD and IDA in the participants in W1 and W2. Prevalence of persistent antenatal CMD (EPDS-V score ≥ 4 in both W1 and W2) was 16.9% (95% CI: 13.1-20.7). In early pregnancy, 9/378 women met criteria for IDA (2.4%, 95% CI: 0.8-3.9), but by late pregnancy 57/378 women had IDA (14.8%, 95% CI: 11.2-18.4). There were 50 new cases at W2, an incidence risk of 13.2% (95% CI: 9.8-16.7). The Hb and ferritin concentration of participants in early (W1) and late (W2) pregnancy are presented in Figure 1.

**Table 2:** Prevalence of maternal common mental disorders and iron deficiency anaemia among 378 pregnant women who provided compete data, Ha Nam province, Viet Nam in 2010.

**Health problem**	Having problem in Wave 1^c^ n (%)	Having problem in Wave 2^d^ n (%)	Having problem in both Waves n (%)
CMD^a^	147 (38.9)	106 (28.0)	64 (16.9)
IDA^b^	9 (2.4)	56 (14.8)	6 (1.6)

^a^ CMD: Common mental disorders (EPDS score ≥ 4); ^b^ IDA: Iron deficiency anaemia (ferritin < 15 ng/ml and Hb < 11 g/dL); ^c^ Early pregnancy (from 12-20 gestational weeks); ^d^ Late pregnancy (last trimester).

### Taking iron supplements

A third of the cohort reported that they had either not taken iron supplements or had taken them for less than 35% of the pregnancy (see Table 3). Of those who took no supplements the main reasons were discomfort from or fear of side effects (16/39, 41.0%), thinking that it was unnecessary (12/39, 30.8%), and having no awareness that they should be taking them (5/39, 12.8%). 

**Table 3:** Duration of taking iron supplements and advice about iron supplementation during current pregnancy among 378 women who provided compete data, Ha Nam province, Viet Nam in 2010.

**Characteristic**	**Number**	**%**
Duration of taking iron supplements		
*Not taking*	39	10.3
*< 35% of duration of pregnancy*	83	22.0
*36-70% of duration of pregnancy*	133	35.2
*>70% of duration of pregnancy*	123	32.5
Who advised to take iron supplements		
*No one*	107	28.3
*Commune health station staff or village health worker*	145	38.4
*Hospital doctors*	37	9.8
*Private doctors or pharmacists*	61	16.1
*Family, relatives, or friends*	81	21.4
*Mass media*	31	8.2

### Structural Equation Model

**Figure 2:**
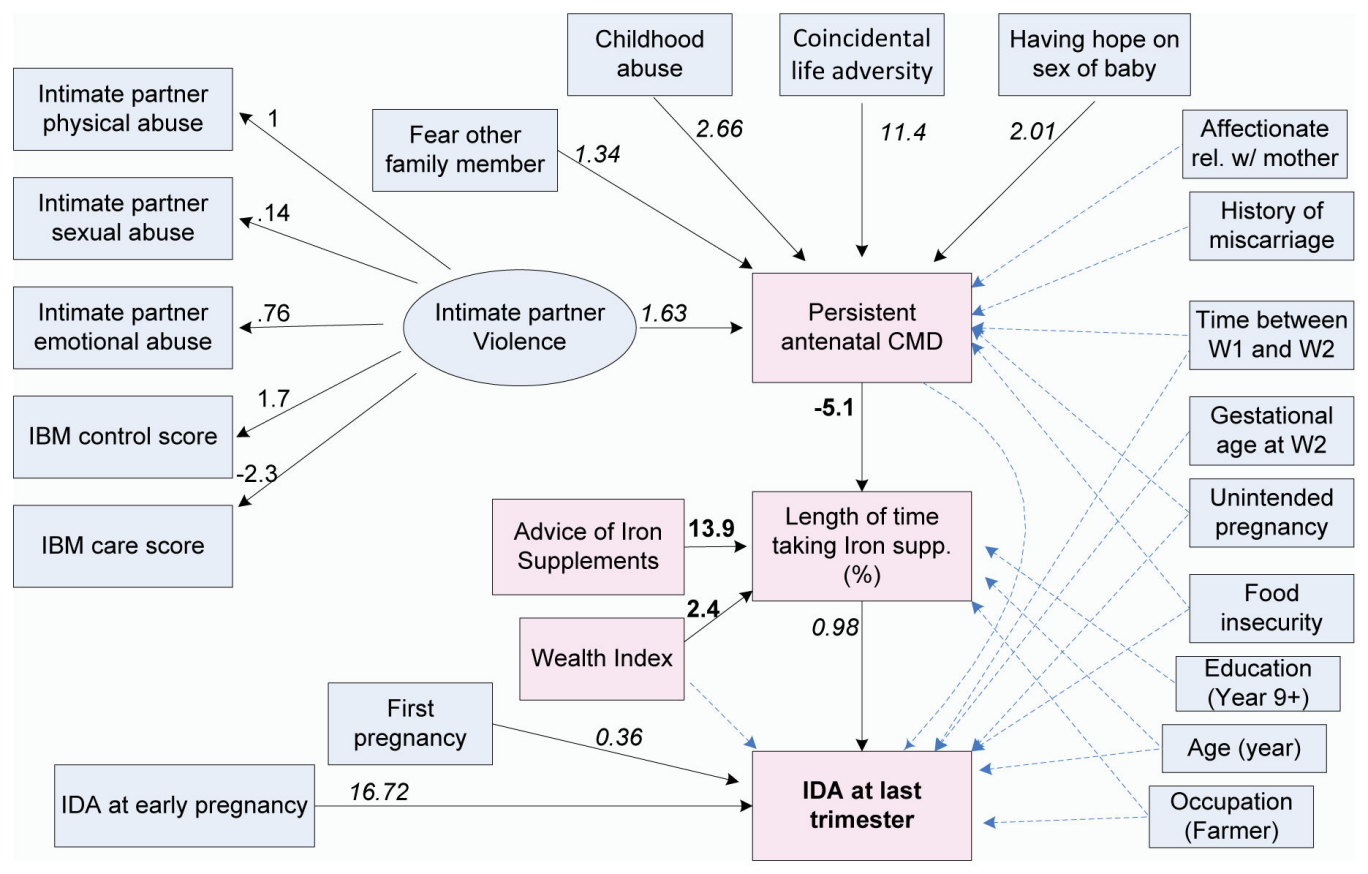
Model predicting IDA in pregnant women during the last trimester, Ha Nam province, Viet Nam in 2010. ∙ All of the variables in the diagram (presented in rectangular boxes) are observed except for the unmeasured (latent) variable ‘Intimate partner violence’ (represented as an ellipse). ∙ The dependent variables are: Persistent CMD (yes/no), Length of time taking iron supplements (continuous – percentage points) and IDA at last trimester (yes/no). ∙ Single-headed solid arrows represent statistically significant directional paths, whereas dashed lines indicate hypothesized but non-significant paths. ∙ The values given for the paths from the latent variable (Intimate partner violence) to the 5 variables assessing the quality of the relationship with the intimate partner (e.g. IBM control score) are factor loadings. ∙ Path coefficients in italics are odds ratio and bold path coefficients are the linear regression coefficients representing the variables with direct relationships with Length of time taking iron supplements.

The model predicting IDA in late pregnancy is shown in Figure 2 and Table 4. Statistical measures assessing how well the model fits the observed data suggest, according to established criteria, that the model fits the data very well. The indirect pathways from persistent CMD, advice about taking iron supplements, and household wealth index to IDA in the last trimester of pregnancy were found through the length of time in which iron supplements were taken (Table 4). . The direct effects of persistent CMD and advice about use of iron supplements on IDA were not significant. There was an increased risk of IDA in late pregnancy in women who were in a second or subsequent pregnancy and women who had IDA in early pregnancy. The direct pathway from IDA in early pregnancy to persistent CMD was not statistically significant.

**Table 4:** Full structural equation model of iron deficiency anaemia in pregnant women during the last trimester, Ha Nam province, Viet Nam in 2010.

Parameter estimates	Model coefficient	95% CI
*Iron deficiency anaemia at last trimester*	*Odds ratio*	
Length of time taking iron supplements (%)	0.98	(0.97 to 0.99)
First pregnancy	0.36	(0.16 to 0.83)
Iron deficiency anaemia at early pregnancy	16.72	(3.46 to 29)
Persistent antenatal CMD	1.09	(0.76 to 1.56)
Food insecurity	0.92	(0.68 to 1.25)
Wealth index	1.10	(0.88 to 1.37)
Age (year)	0.78	(0.37 to 1.64)
Occupation - Farmer	0.86	(0.43 to 1.7)
Time between W1 and W2 (weeks)	1.06	(0.94 to 1.2)
Gestational age at W2 (weeks)	1.04	(0.91 to 1.2)
Unintended pregnancy	0.35	(0.08 to 1.48)
*Length of time taking iron supplements (%)*	*Regression coefficient*	
Persistent antenatal CMD	-5.1	(-9.1 to -1.2)
Advice of iron supplements	13.9	(7.1 to 20.7)
Household wealth index	2.4	(0.7 to 4.1)
Education level - Year 9+	6.8	(-2.0 to 15.6)
Age (year)	-2.7	(-8.9 to 3.5)
Occupation - Farmer	-6.0	(-12.8 to 0.8)
*Persistent antenatal CMD*	*Odds ratio*	
Intimate partner violence index	1.63	(1.24 to 2.13)
Having hope on sex of baby	2.01	(1.14 to 3.55)
Unintended pregnancy	1.50	(0.67 to 3.35)
Coincidental life adversity	11.43	(5.47 to 23.87)
Childhood abuse	2.66	(1.3 to 5.47)
Fear other family member	1.34	(1.13 to 1.6)
Affectionate relationship with mother	0.98	(0.54 to 1.79)
History of miscarriage	1.62	(0.86 to 3.07)
Food insecurity	1.10	(0.87 to 1.39)
Time between W1 and W2 (weeks)	0.98	(0.88 to 1.08)
*Intimate partner violence defined by*	*Factor loading*	
Intimate partner physical abuse	1	N/A
Intimate partner sexual abuse	0.14	(0.12 to 0.16)
Intimate partner emotional abuse	0.76	(0.64 to 0.88)
IBM control score	1.7	(1.0 to 2.4)
IBM care score	-2.3	(-2.7 to -1.9)
*Indirect pathway*	*Odds ratio*	
From “Persistent CMD” to “Iron deficiency anaemia at last trimester” via “Length of time taking iron supplements”	1.8	(1.1 to 2.6)
From “Advice of iron supplements” to “Iron deficiency anaemia at last trimester” via “Length of time taking iron supplements”	0.77	(0.65 to 0.93)
From “Household wealth index” to “Iron deficiency anaemia at last trimester” via “Length of time taking iron supplements”	0.96	(0.93 to 0.99)
Fit indices	Estimates	
χ^2^/*df* (p-value)	165/143 (0.7)	
RMSEA (Probability RMSEA <= .05)	0.02	
CFI	0.96	
TLI	0.95	

Persistent antenatal CMD was significantly predicted by having experienced childhood abuse, intimate partner violence, being frightened of other family members, having a strong preference for the baby’s sex and having coincidental life adversity (Table 4).

## Discussion

This is to our knowledge the first study to investigate and provide evidence that there may be a significant indirect effect of psychological and social factors on late pregnancy IDA among women in a rural setting in a low income country. The data of this study support significant pathways from persistent antenatal CMD advice about taking iron supplements, and household wealth to IDA in late pregnancy via the length of time taking iron supplements. We also confirm the high prevalence of both antenatal CMD [17] and IDA [4] in women in rural Viet Nam. 

Several studies have documented associations between IDA and CMD in both postnatal and non-perinatal populations and all concluded that symptoms of IDA either mimic or contribute to depression [8-12]. In our longitudinal study, we observed a relationship in the opposite direction: that in the context of poverty and social adversity, CMD affects IDA via reducing the likelihood that a woman will take the essential iron supplements needed to protect against IDA in pregnancy. It could be that women with low mood and anxiety are less motivated to attend to preventive health care, particularly taking iron supplements [17,18]. Our findings are not to reject the possible effects of IDA on psychological health, but it could be that these develop over a longer period of time and that the magnitude of the effect during pregnancy was not large enough to be detected by the size of the sample in this study. The relationship between IDA and CMD may be reciprocal. However our data suggest that the direction from CMD to IDA is probably stronger than the opposite effect during pregnancy.

This study confirmed the dose-response relationship between antenatal iron supplementation and the probability of IDA during pregnancy [35]. The proportion of women who took no iron supplements during pregnancy in this study is similar to that reported in other developing countries [36,37]. In addition to CMD, there were significant associations between households of lower socioeconomic status and lack of specific advice about and compliance with taking iron supplements. The important role of education about iron supplementation during antenatal consultations in maximizing compliance has been reported in other low- and lower-middle-income countries [38,39]. Our data confirm the crucial role of this aspect of antenatal care in increasing compliance with the regimen. 

The recommendation of daily iron intake is >50.0 mg/day during the second trimester of pregnancy [40]. Typical dietary iron intake in Viet Nam is low in comparison with this recommendation. Data from the 2010 National Nutrition Survey [41] show that daily dietary iron intake is 12.3 mg. Nakamori et al. [42] found that daily iron intake in postpartum women was 10.2 mg in rural Viet Nam. The consumption of iron-rich foods such as red meat, seafood, and eggs is low in Viet Nam because they are expensive [4]. There have been several attempts to fortify iron in foods for women such as milk [43] and fish sauce [44]. However, no iron fortified food has been provided widely in the population. Taking iron tablets is still the most important source to meet the major increased demands for iron during pregnancy in Vietnamese women. However, these are not subsidised and women have to purchase them, reducing access for the poorest women to this essential micro-nutrient. 

The strengths of the study were that it used a systematic approach to recruit a community-based sample of women in early pregnancy of sufficient size to address the research questions and followed them to late pregnancy to generate high quality biological and psychosocial longitudinal data. The advanced structural equation modeling techniques used in this study allow investigation of both direct and indirect relationships [33]. The limitations of the study included using the EPDS, a screening tool, to assess maternal mental health status. The Vietnamese adaptation of this screening tool was found to have high sensitivity (70.0%) and specificity (73%) against the gold-standard of psychiatrist-administered diagnostic interviews to detect perinatal common mental disorders in this setting [25]. However, there is the possibility that the prevalence of persistent antenatal CMD screened by EPDS in this study was either over- or under-estimated. It is unlikely, though that the measurement error of the EPDS would differ by late-pregnancy IDA status, therefore, the magnitude of the relationship between persistent CMD and late-pregnancy IDA will most likely be under-estimated. Many factors contributed to the attrition rate of this study (15.6%) which could affect the findings in either direction. We have carefully compared the baseline data of the women lost to follow-up with those who provided complete data and there were no significant differences. Therefore, we believe there are no systematic biases due to the loss to follow-up. While having a number of interviewers could have introduced bias, we used several strategies to control and diminish this type of bias in this study. These included using the same group of interviewers for the two waves of data collection, conducting a three-day training course for the interviewers prior to the start of the study, constructing a compulsory standardised procedure to follow for each interview, using an observational supervision system and daily monitoring of data quality, and blinding interviewers to study hypotheses. Furthermore, interviewer biases in this study would be non-differential because all interviewers were blinded to the results of blood tests and psychological tests. 

Our finding of the effect of CMD on IDA in pregnant women is novel. We strongly recommend that in addition to addressing IDA, CMD and the social factors which increase risk of CMD should be considered in antenatal care in Viet Nam and other low- and lower-middle-income countries [45]. Iron supplementation during pregnancy is an effective approach to control IDA, in poor countries. In this study, nearly one third of women reported not being advised to take iron supplements and only one third received direct advice about this from a primary health care worker. It is notable that it was undertaken in a country with a very good primary health care system in which each commune health center provides services to on average of 4000 inhabitants and is staffed with 5 health professionals at least one a full-time doctor and one a full-time midwife. The national iron supplementation program was implemented in Viet Nam in 1997, and is intended to be routine in antenatal care in this area [46]. However, there is neither awareness nor consideration of common mental disorders in the primary healthcare action agenda. Therefore, our research evidence indicates that there is an urgent need to revise health promotion strategies in order to improve the quality of the primary healthcare services for women, especially the content of antenatal care. 

In conclusions, the current framework for IDA control and prevention should be revised to include CMD and its psychosocial determinants: domestic violence and limited gender equity as additional risk factors. At the primary health care level the responsibilities of village health workers/ health volunteers should be expanded to play a key role in promotion of iron supplementation. Furthermore, the expanded role of these primary healthcare workers should include screening for CMD, promotion of mental health and especially to control of domestic violence. 
